# Supplementary material for: Movement patterns of two reintegrated African elephant (Loxodonta africana) herds: transitioning from captivity to free-living
Source: PeerJ. 2024 Jun 6;12:e17535. doi: 10.7717/peerj.17535 (PMC11162612; doi:10.7717/peerj.17535)
Supplement: Supplemental Information 1 [file peerj-12-17535-s001.docx]

| **Elephant** | **Captive facility** | **Reintegration Reserve** | **Reintegration Province** | **Reference** |
| --- | --- | --- | --- | --- |
| 1. Adult male 2. Adult female | Waterberg Safaris (Vaalwater) | Shambala Private Game Reserve | Limpopo Province | (Wentzel & Hay, 2015) |
| 1. Sub-adult female |  |  |  |  |
| 1. Juvenile Male |  |  |  |  |
| 1. Adult Female |  |  |  |  |
| 1. Sub-adult female |  |  |  |  |
| 1. Juvenile female |  |  |  |  |
| 1. Juvenile Female |  |  |  |  |
| 1. Calf Male 2. Calf Male |  |  |  |  |
| 1. Adult male |  | Thula Thula Game Reserve | KwaZulu-Natal |  |
| 1. Adult male | Pilanesberg Elephant Back Safaris | !Khamab Kalahari Reserve | North West Province | Personal communication from Brett Mitchell, December 11, 2015 |
| 1. Adult male |  |  |  |  |
| 1. Adult female |  |  |  |  |
| 1. Adult male |  |  |  |  |
| 1. Adult male |  |  |  |  |
| 1. Calf female |  |  |  |  |
| 1. Juvenile female |  |  |  |  |
| 1. Adult female | Knysna Elephant Park | Gondwana Game Reserve | Western Cape | (Wentzel & Hay,2015) |
| 1. Adult female | Brian Boswell circus & Knysna Elephant Park |  |  |  |
| 1. Adult male |  |  |  |  |
| 1. Adult male | Elephants for Africa Forever |  |  |  |
