# Supplementary material for: Movement patterns of two reintegrated African elephant (Loxodonta africana) herds: transitioning from captivity to free-living
Source: PeerJ. 2024 Jun 6;12:e17535. doi: 10.7717/peerj.17535 (PMC11162612; doi:10.7717/peerj.17535)
Supplement: Supplemental Information 2 [file peerj-12-17535-s002.docx]

| **Characteristics** | **Shambala Private Game Reserves** | **!Khamab Kalahari Reserve** |
| --- | --- | --- |
| **Bioregion:** | **Central bushveld** (Rutherford, Mucina & Powrie, 2006)**:** | **The eastern Kalahari bushveld** (Rutherford, Mucina & Powrie, 2006)**:** |
| **Mean annual precipitation:** | 559mm (highest rainfall from November to March) (Rutherford, Mucina & Powrie, 2006). | 362mm (highest rainfall from October to March) (Rutherford, Mucina & Powrie, 2006). |
| **Soil type:** | This area has dystrophic, acidic sandy, loamy to gravelly soil, characterized by weak structure with a low to medium base status, which reflects a higher effective rainfall than the rest of the savanna region (Rutherford, Mucina & Powrie, 2006). | The soils are mostly red, aeolian sand with a weak structure and a high base status with surface calcrete and silcrete (Rutherford, Mucina & Powrie, 2006). |
| **Vegetation:** | The vegetation is classified as Waterberg Mountain bushveld and is mostly dominated by tall, deciduous *Terminalia service* and *Burkea africana* woodland with a moderate to well-developed grass layer whereas *Diplorhynchus condylocarpon* dominates the rocky hills. Flat areas and lower slopes with more clay than the surrounding sands, are dominated by *Acacia tortilis* and *A. nilotica* [62]*.* The higher slopes are covered with vegetation ranging from *Faurea saligna* to *Protea caffra*. | The vegetation is classified as Molopo Bushveld and features open woodland to closed shrubland with trees with a well-developed grass layer (dominated by *Acacia erioloba, Boscia albitrunca, Lycium cinereum, L. hirsutum and Rhigozum trichotomum*). The shrub layer comprises of creeping species such as *Citrullus lanatus*, *Acanthosicyos naudinianus, and Cucumis africanus L.*, which are important food and water sources for animals. Graminoids include species such as *Aristida meridionalis, Eragrostis lehmanniana, Aristida congesta,* etc. |
| **Animal Species:** | Impala (*Aepyceros melampus*), blue wildebeest (*Connochaetes taurinus*), zebra (*Equus quagga*) and giraffe (*Giraffa camelopardalis giraffe*) as well as predator species (Spotted hyenas (*Crocuta crocuta*), lion (*Panthera leo*) and leopard (*Panthera pardus*). Species such as the white rhinoceros (*Ceratotherium simum*), African buffalo (*Syncerus caffer*) and African elephant (*Loxodonta africana*) are also present on this privately owned reserve. | Lion, leopard, African wild dog (*Lycaon pictus*), cheetah (*Acinonyx jubatus*), spotted and brown hyena (*Parahyaena brunnea*), as well as the typical ungulate species of the Kalahari. The reserve also has several large herbivores such as African buffalo, gemsbok (*Oryx gazella*), common eland (*Taurotragus oryx*), black rhinoceros (*Diceros bicornis*), white rhinoceros (*Ceratotherium simum*) and elephants (*Loxodonta africana*). |
